# From cognitive overload to institutional escalation: formalizing the supply-demand production condition in patient-safety failure

**DOI:** 10.3389/frhs.2026.1846144

**Published:** 2026-09-08

**Authors:** Yngve Mikkelsen

**Affiliations:** Independent Researcher, Jar, Norway

**Keywords:** agency theory, cognitive load, effort-accuracy trade-off, health system governance, institutional escalation, patient safety, physician burnout, physician workforce

## Abstract

Physician exit, unit closures, and regulatory interventions are conventionally treated as separate workforce and governance problems. The workload–patient safety literature documents the empirical relationship between system strain and deterioration in outcomes; the agency literature describes the information asymmetry governing physician behavior; the organizational safety science literature describes how systems drift toward failure under production pressure. What remains insufficiently specified is a formal account of the production conditions that may connect these literatures, and a candidate mechanism through which institutionally determined cognitive demands may translate, via physician decision-making, into outcome deterioration, recursive self-amplification, and observable institutional escalation. This paper offers a formal conceptual specification of that proposed mechanism. We represent clinical outcome quality as a function of the ratio of effective cognitive supply to cognitive demand: O = f(S/D), where S = I · f(WM, e, *λ*) and D = f(v, c, a). The notation is intended to make hypothesized relationships explicit and generate testable predictions; it is not an empirically estimated or validated cognitive production function. When effective supply becomes insufficient relative to demand, the effort-accuracy trade-off is expected to favor resource-conserving reasoning, increasing the risk of outcome deterioration. Institutions may observe indirect proxies of strain but not the proposed latent S/D condition directly or reliably in real time, creating an information asymmetry that can impede correction. A recursive feedback loop with asymmetric updating is proposed to account for nonlinear degradation and recovery. Drawing on Currie and colleagues' (2012) analysis of institutional work in healthcare and Hirschman's exit-voice-loyalty framework, we propose a four-tier escalation model (T1–T4) culminating in the agent's veto. T1–T4 is intended as a typical cost-increasing pathway rather than an invariant sequence; tiers may overlap or be skipped. A 31-case illustrative inventory across 16 jurisdictions (1999–2026) illustrates that phenomena classified as T4 are documented across diverse contexts but does not validate the causal framework or estimate prevalence. Six testable propositions are derived.

## 1 Introduction

Healthcare systems worldwide face a persistent mismatch between the cognitive demands placed on physicians and the capacity available to meet them. Physician burnout is associated with poorer patient-safety outcomes (1), while extended work hours have been associated with serious medical errors (2). Physicians exposed to persistent overload may reduce professional effort and clinical productivity (3, 4), with burnout strongly associated with turnover intention (1). Organizational safety science has described how systems migrate toward failure boundaries under sustained production pressure (5–7). What remains insufficiently specified is the production condition connecting these observations: how system-imposed cognitive demands interact with available physician cognitive capacity, how this affects decision quality, and how persistent imbalance may become visible through institutional escalation.

**Figure 1:**
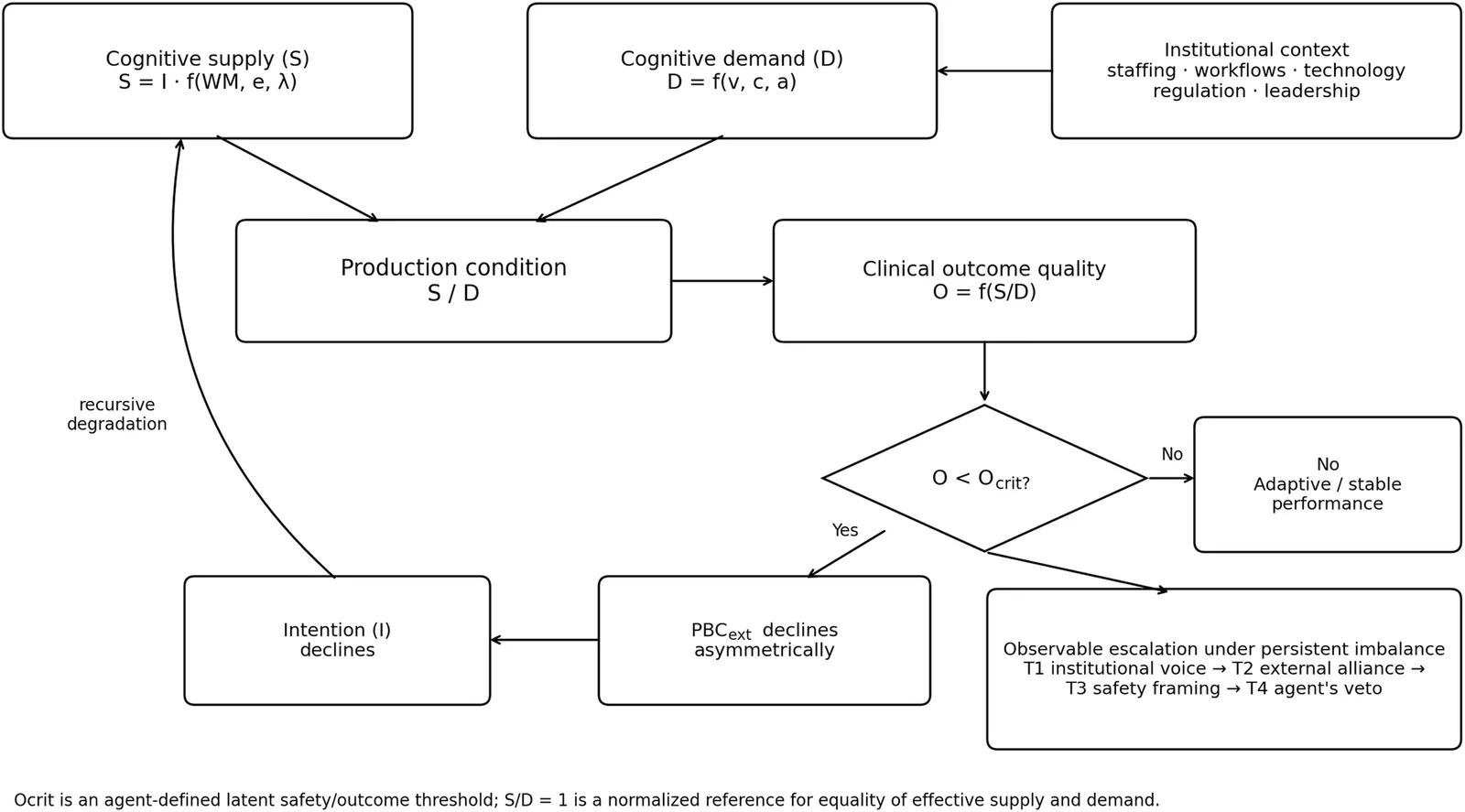
The cognitive supply-demand framework. Effective cognitive supply S = I·f(WM, e, *λ*) and cognitive demand D = f(v, c, a) define the proposed production condition S/D. Outcome quality O is hypothesized to vary nonlinearly with S/D. S/D = 1 is a normalized reference denoting equality of effective supply and demand, not a universal clinical threshold. O_crit_ is a separate agent-defined latent outcome/safety threshold. If O falls below O_crit_, asymmetric updating of perceived behavioral control (PBC_ext_) is hypothesized to reduce intention and effective supply, producing a recursive degradation loop. Persistent unresolved imbalance may generate observable escalation from T1 institutional voice through T4, the agent's veto; this pathway is typical rather than invariant. Construct definitions and candidate measures are summarized in Table 2.

This paper offers a formal conceptual specification of that mechanism. We propose that clinical outcome quality depends on the relationship between effective cognitive supply and cognitive demand, represented as O = f(S/D). The notation is intended to make assumptions explicit, organize existing knowledge, and generate falsifiable predictions; it is not an empirically estimated or validated cognitive production function. When effective supply becomes insufficient relative to demand, the effort-accuracy trade-off (8) is expected to favor resource-conserving reasoning strategies, increasing the risk of outcome deterioration. Because healthcare operates within an agency relationship in which physicians bear personal and professional responsibility for outcomes while institutions substantially influence the conditions determining demand, persistent imbalance may generate an accountability trap and a recursive degradation process.

The institutional consequences may follow a generally predictable pattern. Currie and colleagues (9) examined the institutional work through which medical professionals retain power, demonstrating how they invoke risk and safety arguments to reshape governance structures. Hirschman (10) offered the foundational distinction between exit, voice, and loyalty as responses to organizational decline. Building on both, we introduce a four-stage escalation model: resource arguments through institutional channels (T1), external alliance-building (T2), patient-safety framing (T3), and what we call the agent's veto (T4). This extension draws on agency theory: the principal-institution can influence the factors that determine demand but cannot compel the agent's ongoing participation. T4 therefore represents a proposed structural expression of agency power when prevailing operating conditions are no longer accepted. The T1–T4 pathway may serve as an observable institutional signal of an underlying supply/demand imbalance.

The framework makes three distinct contributions: (a) a proposed cognitive production condition that may connect institutional demands to outcome quality through the effort–accuracy trade-off; (b) an agency-based accountability asymmetry that offers a candidate explanation for why institutions may fail to self-correct; and (c) a tiered escalation model that proposes observable institutional proxies for otherwise hidden cognitive strain.

## 2 The cognitive supply-demand framework

The framework originates from two well-established evaluation traditions. Donabedian's (11, 12) structure-process-outcome model suggests that structural conditions influence clinical processes, which then determine outcomes. Pawson and Tilley's (13) realist evaluation framework states that outcomes result from mechanisms activated within specific contexts. Both approaches do not fully specify how mechanisms translate structure or context into the quality of processes. The cognitive supply-demand framework addresses this gap by proposing the S/D ratio as a candidate production condition through which institutional structure may influence the quality of clinical decisions and, ultimately, patient outcomes. To develop this proposed mechanism, we start with the nature of the input into clinical care: physician effort, understood as medical decision-making within an agency relationship.

### 2.1 Healthcare as agency: effort is the input

Arrow (14) identified healthcare as a classic example of information asymmetry. McGuire (15), in the Handbook of Health Economics, showed that physicians simultaneously serve multiple principals—patients, institutions, insurers, and their profession—and that physician effort is the main input in healthcare production. Mikkelsen (16), through Repertory Grid Technique interviews with 26 physicians across five clinical specialties, empirically demonstrated that physicians perceive clinical quality as the match between available resources and task demands. It also showed that perceived behavioral control is the main mechanism through which enablers and barriers influence physician effort. In this context, effort refers to medical decision-making (15, 16), including diagnosis, treatment choices, risk assessment, and care coordination. Any factor influencing the quality of these decisions impacts the overall outcome. If effort is the input, then the physician's ability to provide that effort—and the system's demands on it—become key variables that determine the quality of the outcome.

### 2.2 Cognitive supply (S)

Cognitive supply refers to the effective processing capacity a physician can deploy for clinical work at a given time. We represent this conceptually as S = I · f(WM, e, *λ*), where WM denotes working-memory capacity (17); e denotes expertise-mediated processing efficiency, including low-cost pattern recognition through illness scripts (18, 19); *λ* denotes fatigue and circadian decline affecting executive function (2, 20); and I represents behavioral intention in the Theory of Planned Behavior (21), reflecting the extent to which available capacity is deployed toward the clinical task. The multiplicative placement of I is a theoretical proposition: it expresses the hypothesis that intention may scale effective deployment of available capacity rather than simply add to it. A physician who has substantially disengaged from the clinical task may therefore deploy substantially reduced effective capacity even when expertise remains intact. This is a testable theoretical prediction, not an established empirical finding, and its magnitude and boundary conditions require empirical investigation.

One illustrative candidate specification is f(WM, e, *λ*) = WM · e · (1—*λ*), with *λ* ∈ [0,1] representing the proportion of capacity lost to fatigue. This expression is not proposed as the true or validated functional form. Its purpose is to make one possible interaction logic explicit: effective supply may depend jointly on available working-memory capacity, expertise-mediated efficiency, and fatigue-related capacity loss. Additive, nonlinear, and alternative interaction structures remain plausible and should be compared empirically. The formalization therefore converts verbal assumptions into contestable hypotheses rather than asserting a measured cognitive law.

Supply-side variables are susceptible to policy influence, but cognitive supply is also socially and contextually embedded. WM is biologically constrained, whereas expertise can be supported through training and appropriate case allocation and fatigue can be influenced through scheduling, rest provisions, and workload distribution. Effective supply may also be shaped by team trust, leadership quality, psychological safety (22), conflict, moral distress, professional support, health, caregiving responsibilities, grief, and other personal-life strain. Some contextual influences may operate through I or *λ*, but the framework does not assume that all such effects are reducible to these pathways; direct effects and additional mediators are plausible. The current individual-level specification is therefore a parsimonious starting point, and extension to team-level and socially embedded cognitive dynamics is an important next step.

### 2.3 Cognitive demand (D)

Cognitive demand refers to the system's requirement for a physician's processing capacity. Following Sweller's (23) cognitive load framework, applied at the system level rather than the instructional level, we define D = f(v, c, a), where v is throughput volume, or the number of patients per unit time, which acts as a demand multiplier with nonlinear effects as utilization approaches capacity (24); c is task complexity, the intrinsic cognitive load influenced by clinical acuity, diagnostic difficulty, and comorbidity burden; and a is system friction, the extraneous cognitive load caused by institutional design choices, including documentation requirements, EHR navigation, alert burden, administrative processes, and poorly designed workflows (25–28).

The decomposition is important for policy. Task complexity (c) is substantially influenced by epidemiological and patient-level factors that are often outside immediate institutional control. Volume (v) can be partly managed through capacity planning. System friction (a) is primarily an institutionally modifiable design parameter, although it is also shaped by regulation, vendor constraints, payment systems, and professional norms. Examples include EHR workflows, documentation requirements, alert thresholds, administrative procedures, and poorly designed work processes (25–28). In this model, complexity refers to inherent clinical challenge, whereas friction refers to extraneous cognitive work generated by the surrounding system. Friction may therefore represent a particularly actionable target for balancing supply and demand, but this is a theoretical proposition to be tested rather than a conclusion established by the illustrative inventory.

The overall cognitive supply–demand framework and its proposed recursive and escalation pathways are summarized in Figure 1.

### 2.4 The outcome function and the effort–accuracy trade-off

We define clinical outcome quality as O = f(S/D). Modeling outcomes as a ratio rather than a difference reflects a specific structural choice: it predicts that equal proportional changes in S and D produce symmetric effects on outcomes. A well-resourced system with high absolute D can fail just as severely as an under-resourced system with moderate D, when each crosses a contextually relevant capacity boundary. When S ≥ D, the physician has enough cognitive capacity for thorough analytical reasoning, and outcomes tend to cluster around the competence-determined optimum O*. When S < D, the physician faces the effort–accuracy trade-off described by Payne, Bettman, and Johnson (8): the adaptive decision-maker switches from accuracy-maximizing to resource-conserving strategies, leading to anchoring, premature closure, availability bias, and other diagnostic errors mapped by Croskerry (18) and Norman and colleagues (29).

We hypothesize that the relationship between outcome quality and S/D may be nonlinear and approximately sigmoid. The value S/D = 1 is used as a normalized theoretical reference point denoting equality between effective cognitive supply and demand; it is not proposed as a universal biological or clinical threshold. The S/D ratio at which clinically meaningful deterioration begins may vary across physicians, tasks, specialties, settings, and outcome measures and therefore requires empirical estimation. The sigmoid form is motivated by analogy with queuing theory, which predicts accelerating degradation as utilization approaches capacity (24), and by the observation that stressed systems may appear stable until performance deteriorates rapidly. It is not formally derived from the preceding equations and is presented explicitly as a testable hypothesis (Proposition 1), not as an established functional relationship.

### 2.5 The recursive feedback loop

The key aspect of the framework is that the S/D ratio changes dynamically. When O falls below a critical outcome threshold, Ocrit, defined by the agent because the physician personally bears professional and ethical responsibility for clinical decisions, we hypothesize that perceived behavioral control (PBC) may update recursively. Ocrit is distinct from the normalized S/D = 1 reference: it is a latent, agent-defined threshold concerning the acceptability or safety of outcomes rather than a fixed supply-demand ratio. Bandura (30) links perceived inefficacy to performance deficits and reduced motivation; conservation of resources theory predicts that initial resource loss can increase vulnerability to subsequent losses (31); and the second-victim literature documents how adverse events may undermine confidence and engagement (32, 33). Blame-oriented responses and weak just-culture protections (34) may plausibly amplify this mechanism, whereas psychologically safe and supportive responses may moderate it (22). These pathways are theoretical extensions requiring empirical testing. A related preprint develops the recursive extension of the Theory of Planned Behaviour in greater detail (35).

The proposed asymmetric updating rule (*φ*^−^ > *φ*⁺) is a distinct theoretical hypothesis rather than a result derived from the preceding equations. It is motivated by work on perceived behavioral control (36), the greater salience and impact of negative than positive events (37), and loss aversion (38), but its magnitude and boundary conditions require empirical estimation. The recursive structure can be described as a difference-equation system. Equations 1–4 treat WM, e, Attitude, and SN as locally constant; D varies on an institutional timescale (policy changes, seasonal volume shifts), and S on a shorter personal timescale through fatigue (*λ*) and the recursive intention mechanism (I).

O(t)=f(S(t)/D(t)) (1)

In words: outcome quality at time t is hypothesized to depend on the current ratio of effective cognitive supply to cognitive demand.

$$\mathrm{PB}C_{\mathrm{ext}}(t+1)\;=\;\mathrm{PB}C_{\mathrm{ext}}(t)\;+\;\varphi \;(O(t)\;\text{–}\;O_{\mathrm{crit}}),\;\mathrm{where}\;\varphi \;=\;\varphi^{\text{-}}\;\mathrm{if}\;O(t)\;<\;O_{\mathrm{crit}},\;\mathrm{and}\;\varphi \;=\;\varphi^{+}\;\mathrm{if}\;O(t)\;\geq \;O_{\mathrm{crit}},\;\mathrm{with}\;\varphi^{\text{-}}\;>\;\varphi^{+}\;>\;0$$ (2)

In words: perceived external behavioral control is updated by the gap between the observed outcome and the agent-defined outcome threshold, with larger downward than upward updating proposed.

$$I(t+1)\;=\;g(\mathrm{Attitude},\;\mathrm{SN},\;\mathrm{PB}C_{\mathrm{ext}}(t+1))$$ (3)

In words: intention at the next time point is proposed to depend on attitude, subjective norms, and the updated perception of external behavioral control.

S(t+1)=I(t+1)f(WM,e,λ(t+1)) (4)

In words: effective cognitive supply at the next time point depends on intention and the physician's available cognitive resources, including expertise and fatigue state.

When O(t) < O_crit_, the term (O(t)—O_crit_) is negative, and *φ*^−^ is the larger multiplier, causing PBC_ext_ to drop sharply. When O(t) ≥ O_crit_, the term becomes positive, but *φ*⁺ is smaller, leading to slower recovery. The constraint *φ*⁺ > 0 ensures that positive outcomes still produce some PBC recovery. The asymmetry *φ*^−^ > *φ*⁺ imparts a ratchet property to this system: degradation accelerates more easily than recovery. It also predicts hysteresis—the D level that triggers collapse is lower than the D level needed for recovery, because PBC_ext_ must be rebuilt through sustained positive results before I and S recover. This aligns with clinical observations that restoring a failed system typically requires much more resources than preventing failure—a prediction that is non-trivial and not derived from verbal theories of burnout or workload strain.

### 2.6 The accountability trap

The recursive loop exists within an agency structure that may impede self-correction. The institution substantially influences factors determining D, including staffing, system design, workload distribution, and technology choices. The physician bears personal, professional, ethical, and sometimes legal responsibility for O. Institutions may have partial proxies for cognitive strain, including overtime, sick leave, incident reports, turnover, or informal feedback, but such indicators are typically indirect, noisy, or lagging and do not provide direct real-time observation of S or the proposed S/D condition. The physician may be better positioned to judge whether current conditions permit safe practice but cannot independently control D. This creates an accountability trap: the agent is held accountable for outcomes shaped partly by conditions the principal manages, while the principal has incomplete information about the agent's capacity to manage those conditions. The information gap is therefore partial rather than absolute, but may still hinder timely institutional correction.

### 2.7 From partially observable strain to institutional escalation: T1–T4

Because the proposed S/D condition is not directly observable in real time and available institutional proxies are incomplete, persistent imbalance may become more visible through institutional escalation. Currie and colleagues (9) demonstrated that medical professionals engage in institutional work to maintain power, invoking risk and patient-safety arguments to reshape governance arrangements when their professional autonomy is threatened. Hirschman's (10) exit-voice-loyalty framework provides the structural logic: actors facing organizational decline can voice concerns, remain loyal, or exit. Combining these perspectives, we propose a four-tier escalation model. T1 involves resource arguments through formal institutional channels (voice within the system). T2 involves professional co-opting and external alliance-building (voice outside the system). T3 involves reframing the issue as a patient-safety crisis, activating regulatory and public accountability mechanisms that are qualitatively different from resource complaints.

We expand this framework with a fourth tier. T4 is what we call the agent's veto—our term for a veto over prevailing operating conditions expressed through individual exit, collective withdrawal, or an organizational reset precipitated by escalation. This addition is based on the underlying agency logic. Institutions may disregard T1, contain T2, or respond to T3, but they cannot compel an agent's continued participation once withdrawal is chosen. This structural dependency gives T4 a qualitatively different character from earlier tiers and marks the point at which the proposed imbalance may produce consequences that are substantially more difficult for the institution to absorb.

The T1–T4 model is intended as a typical cost-increasing escalation pathway, not a strict invariant sequence. Tiers may overlap, stages may be skipped, and escalation may follow non-monotonic paths. For example, a physician may pursue T1 and T2 simultaneously, or a sentinel event may precipitate direct T3 safety framing. Specialty, country, employment model, unionization, litigation risk, leadership, and labor-market conditions may alter the pathway. The theoretical claim is therefore that unresolved imbalance can generate progressively more consequential forms of voice and exit, not that every case must pass sequentially through all four tiers.

Loyalty dynamics are a central theoretical concern rather than a minor limitation. Physicians may remain under persistent S < D without producing visible T1–T4 escalation. In such circumstances, declining intention or effective cognitive supply may produce quiet deterioration while the institution receives little overt warning. The absence of escalation signals therefore does not imply the absence of the underlying production problem; indeed, silent erosion may be one of the most consequential manifestations of the accountability trap. Modelling loyalty and the conditions under which continued adaptation remains safe is an important extension of the framework.

### 2.8 Relationship to organizational safety science and resilience

The cognitive supply-demand framework intersects with, but is not reducible to, organizational safety science and resilience theory. Reason's (5) latent-condition model, Rasmussen's (6) migration-to-the-boundary framework, and Dekker's (7) account of drift into failure explain how systems adapt under production pressure and how local adjustments can move performance toward unsafe boundaries. Safety-II and resilience engineering extend this perspective by emphasizing everyday successful adaptation and the capacity of systems to respond, monitor, learn, and anticipate under expected and unexpected conditions (39, 40).

The S/D framework is complementary rather than substitutive. It proposes an individual-level cognitive production condition that may help specify when adaptive performance becomes insufficient: demand can exceed effective cognitive supply even while clinicians continue to compensate locally. The framework also adds an agency-theoretic accountability mechanism and an escalation pathway linking hidden strain to institutional signals. Conversely, resilience approaches highlight dimensions the present individual-level model does not capture adequately, particularly team coordination, distributed adaptation, organizational learning, and system-level recovery capacity. The proposed hysteresis mechanism is therefore best understood as a testable hypothesis about asymmetric degradation and recovery that is conceptually relevant to resilience, not as a property already established by Safety-II.

### 2.9 Relationship to adjacent models and operationalization

Tables 1, 2 make explicit both the framework's incremental theoretical contribution and the empirical status of its constructs. Table 1 compares S/D with adjacent models; Table 2 identifies candidate measurement approaches. These mappings are illustrative and do not imply that the proposed constructs have yet been validated on a common scale.

**Table 1:** Comparison of the cognitive supply-demand framework with adjacent theoretical models.

Framework	Unit of analysis	Core mechanism	Primary outcome	Dynamic/recursive	Accountability	What S/D adds
JD-R (41)	Worker/job	Balance of job demands and resources	Burnout, engagement, performance	Dynamic but not specified as this feedback system	Not central	Links imbalance to clinical decision production, agency asymmetry, and escalation
Cognitive load theory (23)	Individual cognition	Intrinsic and extraneous load relative to limited capacity	Learning/task performance	Generally task-level	Not central	Extends load logic to clinical production conditions and institutional demand drivers
Rasmussen (6)/drift models	Socio-technical system	Adaptive migration under production pressure	Movement toward safety boundaries/failure	Yes, system adaptation	Indirect	Adds an explicit individual cognitive supply-demand mechanism and agent-principal structure
Agency theory (14, 15)	Principal-agent relationship	Information asymmetry, incentives, non-contractible effort	Effort/allocation/contractual outcomes	Not inherently recursive	Central	Connects agency asymmetry to cognitive capacity, outcomes, and observable escalation
S/D framework	Physician within institutional system	Effective cognitive supply relative to system-imposed demand	Clinical decision quality; loyalty, voice, escalation, exit	Explicit recursive degradation and recovery hypotheses	Central: accountability trap	Integrates cognitive production, agency, and institutional escalation in one testable framework

**Table 2:** Construct definitions, measurement status, and candidate operationalization strategies.

Construct	Conceptual role	Measurement status	Candidate indicators/instruments	Scaling/empirical note
WM	Available working-memory capacity	Proxy-measurable	Working-memory span/updating tasks; task-specific cognitive performance	Biologically constrained; no assumption of a universal clinical unit
e	Expertise-mediated processing efficiency	Latent/proxy-measurable	Training level, specialty experience, validated expertise tasks, diagnostic efficiency	Should reflect efficiency rather than seniority alone
*λ*	Fatigue/circadian capacity loss	Proxy-measurable	Karolinska Sleepiness Scale (42), sleep duration, time awake, shift timing	Candidate specification may normalize λ to 0–1; calibration required
I	Behavioral intention/task engagement	Latent/proxy-measurable	TPB-based intention scales (21), engagement measures, behavioral indicators	Multiplicative scaling is a theoretical hypothesis, not established
v	Throughput volume	Directly measurable	Patients per hour/shift, encounters, messages, orders, task counts	Rate measures should be matched to setting and time window
c	Intrinsic clinical complexity	Proxy-measurable	Acuity, APACHE II (43), Charlson index (44), diagnostic uncertainty, multimorbidity	Instrument must fit specialty; not interchangeable across settings
a	System friction/extraneous load	Proxy-measurable	Time-motion studies (26), EHR click/log data, documentation time, alerts, interruptions	Partly institutionally modifiable; may be externally constrained
D	Aggregate cognitive demand	Latent/proxy-measurable	NASA-TLX (45), task-load measures, models combining v/c/a	Requires validation; raw v, c, a measures are not naturally commensurate
O	Clinical outcome quality	Direct or proxy-measurable depending on outcome	Errors, diagnostic accuracy, process quality, adverse events, risk-adjusted outcomes	Outcome choice affects estimated threshold and functional form
Ocrit	Agent-defined safety/outcome threshold	Latent	Scenario-based elicitation, qualitative interviews, stated-threshold methods	Not directly observable; distinct from S/D = 1
S/D	Proposed production condition	Latent composite	Joint estimation from validated indicators of S and D	Requires normalization, calibration, or latent-variable modelling before numerical interpretation

## 3 Illustrative case inventory: observability of T4

The inventory is an illustrative compilation, not a probabilistic sample; no causal or prevalence claims are intended. Its purpose is limited to illustrating that phenomena classified as T4—the agent's veto—are observable across documented institutional settings. We assembled 31 publicly documented patient-safety incidents across 16 jurisdictions (1999–2026) from peer-reviewed literature, regulatory reports, professional association statements, and quality journalism, using inclusion criteria requiring physician-articulated system-level concerns and documented escalation through at least one tier (Supplementary Material S2). The sampling strategy preferentially captures visible cases and is therefore subject to media visibility, language, jurisdictional, and selection biases.

Within this illustrative inventory, 24 of 31 cases (77%) reached T4. The framework defines three possible veto modes: individual physician exit (T4-I), collective withdrawal or strike action (T4-G), and organizational reset prompted by escalation, such as executive departure, service closure, or major policy reversal (T4-O). In the 31-case inventory, the observed T4 cases comprise one T4-G case and 23 T4-O cases; no T4-I case is present in this purposive sample. Patient-safety framing (T3) was present in all cases and should be understood as a property of the sampling frame rather than an estimate of prevalence. System friction (a) was coded in 84% of cases (*κ* = 1.00), volume (v) in 65%–67% (*κ* = 0.93), and task complexity (c) in 13%–40% (*κ* = 0.37). These percentages characterize the selected cases only. The inventory contains no comparison sample selected independently of escalation status; accordingly, it cannot establish that friction or any other coded demand driver predicts T4. Such inference would require a controlled, matched, or prospective comparative design. The *κ* values are reported as descriptive measures of coding agreement, not as evidence validating the causal framework.

The inventory does not test the S/D framework, estimate the frequency of escalation pathways, or provide confirmatory evidence for the proposed production condition. Its role is purely illustrative: to document the observability of the proposed T4 phenomenon and show that the coding scheme can be applied to heterogeneous documented cases. Supplementary Material S2 comprises a protocol/manual and an accompanying Excel case inventory. The protocol/manual contains the search strategy, inclusion and exclusion criteria, coding definitions and rules, construct-definition table, inter-coder reliability results, and sensitivity analysis; the Excel file contains the 31-case dataset, both coders' tier and demand-driver assessments, T4 basis, and primary-source documentation. Coding was performed manually by two independent coders; Cohen's *κ* calculations were performed in a standard spreadsheet, and no bespoke statistical analysis code was used.

## 4 Propositions

The following propositions are derived from the conceptual framework and are stated as testable predictions. They should be interpreted as hypotheses generated by the formal specification rather than as empirically validated relationships; functional forms, parameter values, and thresholds require prospective estimation.

### 4.1 Proposition 1 (effort-accuracy threshold)

There exists an empirically estimable range of S/D within which increasing imbalance produces measurable outcome degradation. S/D = 1 is a normalized reference denoting equality of effective supply and demand, not a universal fixed clinical threshold. The sigmoid hypothesis predicts nonlinear acceleration of degradation near the empirically relevant capacity boundary, whose location may vary across individuals, tasks, specialties, settings, and outcome measures.

### 4.2 Proposition 2 (recursive degradation)

When S < D is sustained, the recursive feedback loop (O < O_crit_ → PBC_ext_ erosion → I reduction → S reduction) produces accelerating deterioration rather than linear decline. The asymmetry *φ*^−^ > *φ*⁺ predicts hysteresis: recovery requires reducing D below the level that triggered collapse.

### 4.3 Proposition 3 (tier activation)

Persistent unresolved S < D is expected to increase the likelihood of progressively more consequential escalation strategies from institutional voice toward exit. T1–T4 represents a typical cost-increasing pathway; tiers may overlap, be skipped, or occur in non-monotonic order depending on institutional context.

### 4.4 Proposition 4 (friction dominance)

System friction (a), extraneous cognitive load imposed by institutional design, may be the most frequently actionable demand-side driver, because, unlike complexity (c) or volume (v), it is a modifiable design parameter.

### 4.5 Proposition 5 (cost asymmetry)

The institutional cost of addressing S < D increases with each tier. Intervention at T1 (staffing adjustment, workflow redesign) is significantly less expensive than the consequences of T4 (unit closure, emergency recruitment, regulatory intervention, reputational damage).

### 4.6 Proposition 6 (agent-defined threshold)

Ocrit is an agent-defined latent construct reflecting the physician's judgment of the outcome or safety level below which continued practice becomes unacceptable. It is distinct from the normalized S/D reference and cannot be observed directly by the institution. Ocrit could be investigated using qualitative interviews, stated-threshold methods, or scenario-based instruments presenting progressively degraded working conditions and eliciting the point at which physicians judge practice to be unsafe.

Each proposition suggests candidate empirical strategies. Proposition 1 can be tested prospectively by combining cognitive workload measures such as NASA-TLX (45) with clinical outcome data and estimating alternative linear, nonlinear, and threshold functions rather than assuming a single functional form *a priori*. Proposition 2 is suitable for longitudinal panel studies tracking perceived behavioral control and intention after adverse events. Proposition 3 can be assessed through event-history or sequence analysis of escalation patterns, explicitly allowing overlapping and skipped tiers.

Proposition 4 can be tested by comparing demand drivers across institutions using sampling independent of escalation status and by examining whether changes in administrative burden or workflow friction predict changes in cognitive workload. Proposition 5 can be assessed through cost modelling comparing early-tier interventions with downstream T4 consequences. The magnitude and functional form of any friction-escalation or friction-cost relationship should be estimated empirically rather than assigned an unsupported numerical effect size. Proposition 6 can be explored through qualitative, survey, or scenario-based elicitation studies comparing physicians' safety judgments with institutional performance metrics.

## 5 Discussion

The formalization O = f(S/D) → T1–T4 makes four proposed contributions. First, it offers a candidate mechanism connecting the workload–patient safety literature to the workforce withdrawal literature. Both may reflect aspects of the same underlying phenomenon; the S/D framework proposes a candidate production condition that may link them. Second, it proposes the accountability trap as a structural explanation for why health systems may fail to self-correct: institutions influence D but cannot directly observe S, while the agent who may be better placed to judge S bears personal and professional responsibility for O. Third, building on Currie and colleagues' analysis of institutional work and Hirschman's exit–voice–loyalty framework, it proposes a four-tier escalation model culminating in T4—the agent's veto—and uses the 31-case inventory to illustrate that T4-classified phenomena are documented in practice. Fourth, the recursive difference-equation system generates testable predictions—sigmoid degradation, hysteresis in recovery, and the scaling property of intention on supply—that distinguish the framework from purely verbal accounts of burnout or workload strain.

The framework should be distinguished from, and situated alongside, established measurable constructs and theories. JD-R (41) identifies demand-resource imbalance and predicts outcomes including burnout and disengagement. Measures of burnout, fatigue, staffing strain, EHR burden, safety climate, psychological safety, and physician disengagement may capture determinants, correlates, manifestations, or consequences of cognitive supply and demand rather than interchangeable measures of S/D itself. The S/D framework does not replace these constructs. Its proposed incremental value is to specify a candidate cognitive production mechanism linking institutional conditions to clinical decision-making and outcome quality, embed that mechanism within an agency-theoretic accountability structure, and connect persistent hidden imbalance to observable institutional escalation. The framework therefore proposes one common generative pathway through which some established associations may arise, while allowing that these constructs can also affect outcomes through mechanisms outside S/D.

Physicians may also leave for financial, career-stage, or labor-market reasons unrelated to cognitive overload. The framework does not claim that S < D is the sole reason for exit but suggests it as the mechanism through which exit may cluster around conditions of documented system strain rather than being randomly distributed across institutional contexts. The framework would be disproven by systematic evidence showing institutional escalation to T4 in the absence of workload-related safety concerns, or conversely, by sustained S < D conditions that do not lead to outcome deterioration or escalation. Several features of the framework are individually testable. The sigmoid-threshold claim (Proposition 1) would be disproven by evidence that outcome degradation under increasing cognitive load is linear rather than nonlinear, or that no identifiable threshold exists. The asymmetric updating claim (Proposition 2) would be disproven by evidence that perceived behavioral control recovers as easily after system failures as it diminishes, or that adverse outcomes do not systematically reduce physician intention. The T1–T4 sequence (Proposition 3) would be disproven by evidence that escalation does not follow a cost-increasing pattern, or that T4 events occur without prior voice-based escalation. The accountability trap would be disproven by evidence that institutions can reliably observe S/D in real time and adjust D accordingly before escalation occurs.

The framework is conceptual and explanatory. The equations formalize hypothesized relationships so that their assumptions can be examined and tested; they are not empirically estimated cognitive laws. Apart from the deliberately illustrative candidate specification in §2.2, we do not claim validated functional forms, threshold values, or decay rates. Alternative specifications should be compared empirically. The 31-case inventory is purposeful and subject to visibility, language, jurisdictional, and high-salience selection biases. The framework currently models the individual physician as the unit of cognitive supply; extension to team-level cognition and the broader resilience perspective emphasized by Woods and Cook (46) and resilience engineering (39, 40) is necessary. The T1–T4 model is typical rather than invariant, and loyalty may produce clinically important deterioration without observable escalation. These limitations define a research programme rather than empirical confirmation of the theory.

## 6 Conclusions

When physicians withdraw from clinical practice, institutional responses often focus on recruitment, retention incentives, or individual resilience. The framework developed here suggests that these responses may miss an underlying production problem when effective cognitive demand persistently exceeds supply. The cognitive supply-demand framework offers a formal conceptual connection between institutional demands and clinical decision quality, places that proposed connection within an agency relationship that may impede self-correction, and proposes a tiered escalation model with testable predictions. The illustrative 31-case inventory illustrates that phenomena classified as T4 can be identified across diverse documented contexts but does not validate the causal model. The propositions and operationalization strategies offered here are intended to support prospective measurement, comparison of alternative functional forms, and empirical estimation of the conditions under which imbalance produces deterioration, recovery, loyalty, voice, or exit.

## Data Availability

The original contributions presented in the study are included in the article/Supplementary Material, further inquiries can be directed to the corresponding author/s.
