# Supplementary material for: From cognitive overload to institutional escalation: formalizing the supply-demand production condition in patient-safety failure
Source: Front Health Serv. 2026 Sep 8;6:1846144. doi: 10.3389/frhs.2026.1846144 (PMC13593859; doi:10.3389/frhs.2026.1846144)
Supplement: Supplementary file 1 [file Datasheet1.pdf]

Supplementary Material 1

Self-contained specification of the recursive extension of the Theory of Planned Behaviour

S1. Standard TPB structure

Following Ajzen (1991), behavioural intent at time t is determined by:

```
I(t) = f(A, SN, PBC(t))
```

where A is attitude toward the behaviour, SN is subjective norm, and PBC is perceived behavioural control. Intent is the proximal determinant of behaviour B(t), and behaviour produces an outcome O(t). In the standard model, A and SN are treated as relatively stable within a given behavioural domain.

S2. Two facets of PBC

Ajzen (2002) distinguishes two facets of PBC:

Internal PBC (self-efficacy): confidence in one's own abilities and skills. A physician's belief that they possess the clinical knowledge and competence to manage a given case.

External PBC (controllability): beliefs about whether external factors will permit successful action. A physician's belief that the system—staffing, equipment, time, processes—will support the delivery of safe care.

This distinction is central because the two facets generate different predictions about how outcomes update intent. If feedback operates through internal PBC, adverse outcomes reduce self-efficacy (the learning mechanism). If feedback operates through external PBC, adverse outcomes update beliefs about what the system will support (the context mechanism).

S3. Empirical basis for the updating pathway

The following empirical evidence, drawn from Mikkelsen (2013; DBA thesis, Cranfield University) and formalised in Mikkelsen (2026; under review at BMC Psychology), supports the claim that outcome feedback operates primarily through external PBC under resource-constrained conditions:

(a) Repertory Grid Technique interviews with 26 physicians across five clinical specialities generated 213 personal constructs. External PBC constructs (resource availability, time,

continuity, cooperation, resource utilisation) accounted for 26.3% of all constructs. Internal PBC constructs (interpretation, experience, competency level, confidence) accounted for 16.4%. The external-to-internal ratio was 1.6:1, robust across sensitivity analyses (range 1.1:1 to 2.6:1).

(b) In semi-structured interviews, physicians predominantly attributed adverse outcomes to external factors (time constraints, equipment shortages, staffing levels, system processes). When asked what they would do differently if constraints were removed, physicians consistently reported they would do 'more of the same'—indicating intact self-efficacy.

(c) Negative affect arising from adverse outcomes was directed at 'the whole system' rather than at self-competence.

Conclusion: the primary updating target for adverse outcome experience is external PBC. Internal PBC is present but secondary.

S4. Outcome-dependent updating rule

Let O(t) denote the clinical outcome at time t and O_crit denote the physician's internal reference standard for acceptable performance—the agent-defined threshold below which the recursive loop activates (corresponding to O_crit in the main text; distinct from O*, the competence-determined optimum). O_crit is treated as stable across decision cycles. PBC is updated between cycles as:

$$PBC\_ext(t+1) = PBC\_ext(t) + \varphi \cdot (O(t) - O\_crit)$$

where φ is an updating coefficient that depends on the direction of the discrepancy:

$$\varphi = \varphi^- \quad \text{when } O(t) < O\_crit \quad \text{(adverse outcome)}$$

$$\varphi = \varphi^+ \quad \text{when } O(t) \geq O\_crit \quad \text{(acceptable outcome)}$$

The critical asymmetry condition is:

$$\varphi^- > \varphi^+ > 0$$

That is, an adverse outcome of a given magnitude updates PBC more strongly than a positive outcome of the same magnitude. Degradation of perceived control proceeds faster than its recovery. The constraint $\varphi^+ > 0$ ensures that positive outcomes do produce some PBC recovery; without it, the system would degrade irreversibly. This asymmetry is supported by three

independent literatures: (a) Baumeister et al. (2001), who documented the 'bad is stronger than good' principle across multiple psychological domains; (b) Kahneman and Tversky (1979), whose prospect theory demonstrates asymmetric valuation of losses versus gains; and (c) Hobfoll (1989), whose conservation of resources theory predicts that resource loss is more salient than resource gain.

S5. The recursive system

Substituting the updating rule into the TPB structure yields the following difference-equation system:

```
O(t)         = f(S(t) / D(t))

PBC_ext(t+1) = PBC_ext(t) + φ · (O(t) − O_crit)

I(t+1)       = g(A, SN, PBC_ext(t+1))

S(t+1)       = I(t+1) · f(WM, e, λ(t+1))
```

where:

- O(t) is clinical outcome quality, a function of the supply–demand ratio
- $PBC_{ext}(t+1)$ is updated external perceived behavioural control
- I(t+1) is behavioural intention at the next cycle
- S(t+1) is cognitive supply at the next cycle, where I multiplies capacity (see main text Section 2.2)
- A and SN are treated as approximately constant within a clinical domain
- D is determined exogenously by the institution (staffing, volume, friction)

The system is closed: outcomes at time t shape PBC at t+1, which shapes intent, which shapes supply, which shapes the next outcome. The asymmetry $\varphi^{-} > \varphi^{+}$ gives the system a ratchet property: degradation accelerates more readily than recovery.

S6. Dynamic properties

S6.1 Stability under S ≥ D

When $S \geq D$, outcomes cluster around O_crit or above. The updating term $\varphi^+ \cdot (O(t) - O\_crit)$ is small and positive, producing gradual PBC maintenance or modest increase. I remains stable. S remains stable. The system is in equilibrium.

S6.2 Degradation under sustained S < D

When $S < D$ is sustained, O falls below O_crit. The updating term $\varphi^- \cdot (O(t) - O\_crit)$ is negative and large (because φ^- is large). PBC_{ext} falls. I falls. Because I multiplies capacity ($S = I \cdot f(WM, e, \lambda)$), S falls. The S/D ratio worsens. O deteriorates further. The loop self-amplifies.

S6.3 Hysteresis

Suppose the institution reduces D (e.g., by hiring staff or reducing friction) after a period of sustained $S < D$. Even if D falls below the level that originally triggered the degradation, PBC_{ext} has already been eroded. Because recovery requires sustained $O \geq O\_crit$ to rebuild PBC through the weak φ^+ channel, and because low I means low S means O may remain below O_crit even at the new lower D, the system may remain trapped in the degraded state. The D level required for recovery is therefore lower than the D level that triggered collapse. This is hysteresis: the path into degradation is not simply reversed by removing the original cause.

S7. Worked numerical example

The following illustrative example demonstrates the system's behaviour over five cycles under sustained $S < D$. Parameter values are chosen for transparency, not calibrated to empirical data.

Assumptions:

- O_crit = 0.90 (physician's agent-defined threshold)
- $\varphi^- = 0.30$ (adverse updating strength)
- $\varphi^+ = 0.10$ (positive updating strength)
- Initial PBC_ext = 0.90
- $I = 0.5 + 0.5 \cdot PBC\_ext$ (simplified linear intention function)
- $S\_cap = f(WM, e, \lambda) = 1.0$ (capacity held constant for simplicity)
- $S = I \cdot S\_cap = I$

• D = 1.10 (sustained demand exceeding initial supply)

• O = min(1.0, S/D) (capped at 1.0)

t	PBC_ext	I	S	D	S/D	O	O–O_crit
0	0.90	0.95	0.95	1.10	0.864	0.864	–0.036
1	0.889	0.945	0.945	1.10	0.859	0.859	–0.041
2	0.877	0.938	0.938	1.10	0.853	0.853	–0.047
3	0.863	0.931	0.931	1.10	0.847	0.847	–0.053
4	0.847	0.924	0.924	1.10	0.840	0.840	–0.060
5	0.829	0.915	0.915	1.10	0.832	0.832	–0.068

Interpretation: Over five cycles, PBC_{ext} falls from 0.90 to 0.829. Intention falls from 0.95 to 0.915. Supply falls correspondingly. The outcome gap (O – O_crit) widens from –0.036 to –0.068, demonstrating the self-amplifying nature of the loop. Each cycle's adverse outcome feeds into a larger PBC reduction in the next, because the worsening O produces a larger negative (O – O_crit) term multiplied by the strong φ^- coefficient.

Hysteresis demonstration: Now suppose at t=5 the institution reduces D from 1.10 to 1.00. With S = 0.915 and D = 1.00, S/D = 0.915 and O = 0.915 > O_crit = 0.90. The positive updating fires: $PBC_{ext}(6)$ = 0.829 + 0.10 × (0.915 – 0.90) = 0.829 + 0.0015 = 0.831. Recovery is negligible. PBC was eroded by 0.071 over five adverse cycles but recovers by only 0.002 in one positive cycle. At this rate, restoring PBC to its original level would require approximately 35 positive cycles—demonstrating hysteresis. If D had been reduced to 1.00 at t=0 instead, the adverse loop would never have activated.

S8. Boundary conditions and limitations

(a) The updating rule treats O_crit as stable. In practice, physicians may adjust their reference standards downward under sustained adversity (normalisation of deviance), which would attenuate the loop. This represents a potential moderating factor not included in the present specification.

(b) The model assumes PBC_{ext} updates monotonically in response to outcomes. In reality, a single highly salient event (e.g., a patient death attributed to system failure) may produce a step-change in PBC rather than incremental updating.

(c) The empirical basis is cross-sectional. The temporal dynamics implied by the difference equations have not been tested longitudinally. The propositions on degradation and hysteresis are theoretical predictions that require empirical evaluation through panel designs or natural experiments.

(d) The finding that external PBC is the primary updating target is one of predominance, not exclusivity. Internal PBC (self-efficacy) operates as a secondary pathway, particularly among less experienced physicians.

References

Ajzen I. The theory of planned behavior. Organ Behav Hum Decis Process. 1991;50(2):179–211.

Ajzen I. Perceived behavioral control, self-efficacy, locus of control, and the theory of planned behavior. J Appl Soc Psychol. 2002;32(4):665–683.

Baumeister RF, Bratslavsky E, Finkenauer C, Vohs KD. Bad is stronger than good. Rev Gen Psychol. 2001;5(4):323–370.

Hobfoll SE. Conservation of resources: a new attempt at conceptualizing stress. Am Psychol. 1989;44(3):513–524.

Kahneman D, Tversky A. Prospect theory: an analysis of decision under risk. Econometrica. 1979;47(2):263–291.

Mikkelsen Y. Exploring physicians' decision making and perception of quality in health care delivery [DBA thesis]. Cranfield: Cranfield University; 2013.

Mikkelsen Y. How clinical outcomes reshape physician intent: a recursive extension of the Theory of Planned Behaviour using repertory grid technique. BMC Psychol. Under review. Preprint available at doi:10.31234/osf.io/g8uef_v1
