# Supplementary material for: From cognitive overload to institutional escalation: formalizing the supply-demand production condition in patient-safety failure
Source: Front Health Serv. 2026 Sep 8;6:1846144. doi: 10.3389/frhs.2026.1846144 (PMC13593859; doi:10.3389/frhs.2026.1846144)
Supplement: Supplementary file 2 [file Supplementaryfile1.pdf]

Supplementary Material 2

Search protocol, coding manual, and case inventory

Part A: Search protocol

A1. Objective

To compile a purposive inventory of publicly documented patient-safety episodes in which physician-articulated system-level concerns led to institutional escalation through one or more tiers of the proposed framework (T1–T4).

A2. Source types

Cases were identified through iterative searches across four source types: (1) peer-reviewed academic literature (PubMed, Google Scholar); (2) medical and general news reporting (Google News); (3) professional association statements and reports (e.g., BMA, AMA, medical union publications); and (4) government or regulatory inquiries (national health service reports, parliamentary inquiries, regulatory findings).

A3. Search terms

Initial English-language searches used terms including: "physician resignation workload," "doctor strike patient safety," "hospital unit closure staffing," "healthcare workforce withdrawal," "physician exit unsafe conditions," "maternity safety inquiry," "emergency department overcrowding physician," and "healthcare-associated infection staffing." Norwegian-language searches used equivalent terms for Scandinavian cases. French-language searches were used for Canadian cases.

A4. Snowball and secondary sources

Reference lists and media coverage of included cases were used for snowball sampling. Secondary syntheses were consulted to reduce regional blind spots: Ciapponi et al. for Latin America; Cao et al. for China.

A5. Candidate pool and selection

The initial identification pool comprised 78 candidates. Of these, 31 met the inclusion criteria and were retained in the final inventory, spanning 16 jurisdictions and covering the period 1999–2026. The remaining 47 were excluded for insufficient documentation of escalation tiers

(n = 29), primary driver being individual misconduct rather than system-level workload or governance failure (n = 12), or duplication with included cases (n = 6).

A6. Inclusion criteria

(i) Physician-led or physician-endorsed public articulation of system-level workload or safety concerns; (ii) sufficient documentation to determine whether at least one tier of escalation (T1–T4) occurred; (iii) a clear link in the source material between the raised concern and either clinical performance constraints, patient safety risk, or inability to sustain practice under prevailing conditions.

A7. Exclusion criteria

(i) Cases driven primarily by individual misconduct, purely personal circumstances, or isolated interpersonal conflict; (ii) cases lacking verifiable documentation beyond uncorroborated allegations.

A8. Iterative refinement

The tier schema and demand-driver categories were refined through five documented rounds of re-coding. Each round is recorded in the audit trail columns of the accompanying dataset (xlsx file). The coding approach followed an abductive logic consistent with theory-building case research (Eisenhardt, 1989; Gioia et al., 2013).

Part B: Coding manual

B1. Escalation tiers: operational definitions

Tier	Label	Operational definition	Coding rule
T1	Resource arguments	Physician or medical staff communicate a supply–demand imbalance through legitimate institutional channels: departmental meetings, written memos, incident reports, conversations with management, formal grievance procedures.	Code T1 present when the source record documents a formal or semi-formal communication from clinicians to institutional leadership about resource, staffing, or workload concerns. Generic media references to 'staffing shortages' without documented internal communication are insufficient.
T2	Professional co-opting	Physicians seek allies outside the immediate institutional hierarchy: professional associations, medical unions, media, political representatives, peer networks.	Code T2 present when the source documents engagement with external bodies (unions, professional associations, media) on behalf of the clinical concern. Code conservatively: mere media coverage of an event does not constitute T2

			unless clinicians actively sought that coverage.
T3	Patient-safety framing	The supply–demand imbalance is reframed as a patient-safety crisis, invoking regulatory, legal, or public accountability mechanisms qualitatively different from resource complaints.	Code T3 present when the source documents an explicit statement that patient safety is at risk, patient harm has occurred, or conditions are unsafe. This may come from clinicians, regulators, inquiry reports, or formal investigations.
T4	Agent's veto	The most costly and difficult-to-reverse endpoint: exercise of a veto over existing operating conditions through withdrawal. Three modes: T4-I (individual exit), T4-G (collective withdrawal/strike), T4-O (organisational reset: executive departure, service closure, major policy reversal).	Code T4 present when the source documents: (a) physician resignation, relocation, or refusal to staff linked to the episode (T4-I); (b) collective action, strike, or coordinated withdrawal (T4-G); or (c) executive departure, service closure, or major policy/governance change triggered by the episode (T4-O). T4 requires a paired veto_type code (I, G, or O).

B2. Demand drivers: operational definitions

Code	Label	Operational definition	Coding rule
v	Volume	Patient throughput exceeds the system's capacity to process safely. Includes ED overcrowding, unsustainable patient-to-physician ratios, and bed/staffing shortages that reduce effective capacity.	Code v present when the source explicitly describes patient numbers, caseload, or throughput as a contributing factor (e.g., 'unsustainable patient numbers,' 'overcrowding,' 'insufficient staffing to meet demand').
c	Complexity	Intrinsic clinical complexity of the patient population. Includes increasing case acuity, multi-morbidity, diagnostic difficulty, and the shift of simpler cases to outpatient settings.	Code c present when the source explicitly describes clinical complexity, case acuity, or diagnostic difficulty as a contributing factor. Code conservatively: many cases initially appearing complex on inspection involve friction (governance failures, escalation breakdowns, process deficiencies) rather than irreducible clinical complexity. The friction–complexity boundary is the most judgment-dependent coding decision in this inventory ($\kappa = 0.37$).
a	Friction	System-generated, non-clinical cognitive demands: documentation burden, EHR navigation, alert fatigue, administrative tasks, poorly designed workflows, governance failures, escalation breakdowns, and process deficiencies that consume processing capacity without improving clinical performance.	Code a present when the source describes institutional design or process failures as contributing factors (e.g., 'EHR burden,' 'documentation demands,' 'governance failure,' 'communication breakdown,' 'escalation failure'). Note: the primary coder reclassified several cases from c to a during iterative

			coding, guided by the framework's distinction between intrinsic and extraneous load. This introduces a circularity acknowledged in the manuscript.

B3. Veto type codes

Code	Label	Definition
I	Individual exit	Physician(s) resign, relocate, or refuse to staff the unit, linked to the episode.
G	Group/collective withdrawal	Collective withdrawal, strike action, or coordinated refusal to staff by multiple physicians, linked to the episode.
O	Organisational reset	Executive departure, service closure/reconfiguration, or major policy reversal triggered by the episode.

B4. Coding procedure

(1) For each case, the primary coder reviewed all available source documentation (inquiry reports, regulatory findings, news reporting, professional association statements). (2) Tier assignments were anchored to observable events in the source record (e.g., formal complaint submitted = T1; public safety statement = T3; resignation documented = T4-I). (3) Demand drivers were coded as present when the source record explicitly described the driver as a contributing factor. Multiple drivers could be coded per case. (4) Where cases contained conflicting accounts, coding privileged contemporaneous primary documents and official reports over later commentary. Uncertainty was recorded in the audit trail. (5) A second independent coder reviewed all 31 cases for tier assignments and demand-driver coding.

B5. Inter-coder reliability

Element-wise Cohen's kappa for individual tiers: T1 $\kappa = 0.84$ (strong), T2 $\kappa = 0.45$ (moderate), T3 $\kappa = 1.00$ (perfect), T4 $\kappa = 0.90$ (strong). Exact-match kappa for the full tier pattern: $\kappa = 0.73$ (substantial). For demand drivers: volume (v) $\kappa = 0.93$ (strong), system friction (a) $\kappa = 1.00$ (perfect), task complexity (c) $\kappa = 0.37$ (fair). The low κ for complexity reflects residual ambiguity at the friction–complexity boundary in retrospective case coding.

B6. Sensitivity notes

Coder 2 assigned T2 in four additional cases and complexity (c) in eight additional cases where the primary coder had coded these as absent or reclassified as friction. The primary coder's

reclassification of complexity as friction in several cases was guided by the framework under development, introducing a circularity that is acknowledged in the manuscript. An alternative coding retaining Coder 2's complexity assignments yields friction in approximately 74% of cases and complexity in approximately 39% (vs. 84% and 13% for the primary coder).

Part C: Construct definition table

Supplementary Table S1 (provided as a separate document) defines the three demand-side constructs (v, c, a) used in the cognitive supply–demand framework, with illustrative examples, non-examples, and boundary cases to guide coding and future operationalisation. The table includes a five-step coding decision rule applied during the inventory coding process and a note on inter-coder reliability at the friction–complexity boundary.

Part D: Case inventory dataset

The 31-case inventory dataset is provided in the accompanying Excel file (SM2_case_inventory.xlsx). The dataset contains 16 columns including both Coder 1 and Coder 2 assessments for tiers and demand drivers, primary source URLs, and T4 basis documentation.
