# Supplementary material for: From cognitive overload to institutional escalation: formalizing the supply-demand production condition in patient-safety failure
Source: Front Health Serv. 2026 Sep 8;6:1846144. doi: 10.3389/frhs.2026.1846144 (PMC13593859; doi:10.3389/frhs.2026.1846144)
Supplement: Supplementary file 3 [file Supplementaryfile2.pdf]

Supplementary Table S1. Construct definitions for demand-side components

This table defines the three demand-side constructs (v, c, a) used in the cognitive supply–demand framework, with illustrative examples, non-examples, and boundary cases to guide coding and future operationalisation. The boundary cases column identifies scenarios where classification requires judgement, and explains the reasoning applied in this study.

Construct	Definition	Illustrative examples	Non-examples	Boundary cases and coding guidance
**Throughput volume (v)**<br>*Demand multiplier*	Patient numbers per unit time. Operates as a demand multiplier with nonlinear properties as utilisation approaches capacity. Partially controllable through capacity planning and patient flow management.	• Emergency department overcrowding beyond staffed capacity<br>• Seasonal surge (e.g., winter respiratory admissions)<br>• Pandemic-driven patient volume spikes<br>• Elective surgery backlog creating compressed scheduling<br>• On-call physician covering multiple wards simultaneously	• High complexity with normal volume (code as complexity, not volume)<br>• Administrative burden increasing time per patient without changing patient numbers (code as friction)<br>• Staffing reductions that increase volume per remaining physician (code as volume)	Volume with friction interaction: A department has normal patient numbers but a new EHR system doubles documentation time per patient, effectively halving throughput capacity. Code as friction (a), not volume (v), because the patient count has not changed; the constraint is system-imposed time per case.<br>Volume with complexity interaction: A trauma centre receives a mass casualty event. Code as volume (v) even though individual cases may be complex, because the primary demand driver is the number of simultaneous patients exceeding capacity.
**Task complexity (c)**<br>*Intrinsic cognitive load*	The intrinsic cognitive load determined by clinical acuity, diagnostic difficulty, and comorbidity burden. Largely determined by epidemiological factors outside institutional control. Refers to the inherent difficulty of the clinical problem itself.	• Rare or atypical disease presentation requiring extended differential diagnosis<br>• Multi-organ failure requiring simultaneous management of competing physiological priorities<br>• Diagnostic uncertainty in undifferentiated presentations (e.g., chest pain with atypical features)	• Routine appendectomy in an otherwise healthy patient (low complexity even in a busy unit)<br>• Difficulty caused by a poorly designed clinical pathway rather than by the clinical problem itself (code as friction)<br>• Difficulty caused by insufficient training for the case type (supply-side issue: expertise,	Complexity versus friction: A physician struggles with a patient because the EHR requires 47 clicks to order a standard medication regimen. The clinical decision is straightforward; the difficulty is system-imposed. Code as friction (a), not complexity (c).<br>Complexity versus expertise: A junior physician finds a case

Construct	Definition	Illustrative examples	Non-examples	Boundary cases and coding guidance
		• Paediatric patient with multiple comorbidities and no established diagnosis<br>• End-of-life decision-making involving clinical, ethical, and family dynamics	not demand-side complexity)	difficult that a senior consultant would handle routinely. The intrinsic complexity is low; the difficulty reflects the physician's expertise level (e, supply-side). Code the demand-side construct based on the case's intrinsic properties, not the treating physician's experience.<br>True complexity: An elderly patient presents with chest pain, renal impairment, and anticoagulation, requiring simultaneous assessment of cardiac, renal, and haematological risks with competing treatment implications. Code as complexity (c).
**System friction (a)**<br>*Extraneous cognitive load*	Extraneous cognitive load imposed by institutional design choices. Entirely a modifiable design parameter. Includes documentation requirements, EHR navigation, alert burden, administrative processes, and poorly designed workflows. The defining feature: friction is avoidable load generated by system design, not by clinical necessity.	• Excessive EHR documentation requirements beyond clinical necessity<br>• Alert fatigue from poorly calibrated clinical decision support<br>• Prior authorisation processes requiring physician time for administrative approval<br>• Incompatible IT systems requiring manual data re-entry across platforms<br>• Mandatory non-clinical meetings during peak clinical hours<br>• Redundant documentation for regulatory compliance with no clinical value	• Time spent on genuinely complex clinical reasoning (code as complexity)<br>• Time spent communicating with patients about difficult diagnoses (complexity or clinical effort, not friction)<br>• Workload from high patient volume (code as volume, not friction, even if it feels burdensome)	Friction versus complexity at the boundary: A hospital implements a new sepsis screening protocol that requires physicians to complete a 15-field checklist for every febrile patient. For genuine sepsis cases, the checklist supports clinical reasoning (not friction). For the majority of febrile patients who do not have sepsis, the checklist adds cognitive load without clinical benefit. Code the protocol as friction (a) when applied indiscriminately; the clinical reasoning involved in actual

Construct	Definition	Illustrative examples	Non-examples	Boundary cases and coding guidance
				sepsis management is complexity (c).<br>Friction from institutional response to complexity: A hospital responds to medication errors by adding three mandatory verification steps. The errors reflected genuine complexity; the verification steps are institutional friction. Code the verification burden as friction (a), even though its origin was a complexity-related problem.

Coding decision rule

When classifying a demand driver in the 31-case inventory, the following decision rule was applied:

1. Is the primary driver the number of patients exceeding staffed capacity? If yes → volume (v).
2. Is the primary driver the inherent clinical difficulty of the cases, independent of system design? If yes → complexity (c).
3. Is the primary driver an avoidable institutional design choice that could be changed without altering the clinical task? If yes → friction (a).
4. If the case involves multiple drivers, code all that apply. The constructs are not mutually exclusive.
5. If ambiguity remains after applying rules 1–3, default to friction (a) when the load could have been reduced by institutional redesign, and to complexity (c) when the load is inherent to the clinical presentation. This asymmetric default reflects the framework's prediction that what appears as complexity often functions as friction (see main text, Section 2.3).

Note on inter-coder reliability at the friction–complexity boundary

The low inter-coder agreement for task complexity ($\kappa = 0.37$) reflects residual ambiguity at the friction–complexity boundary in retrospective case coding. In several cases, one coder classified the demand driver as friction (institutional design imposing unnecessary load) while the other classified it as complexity (inherently difficult clinical work). The disagreements clustered around cases where institutional responses to genuine complexity created secondary friction (e.g., mandatory checklists introduced after adverse events). The coding decision rule above was developed iteratively during the coding process to resolve such cases. Prospective studies using real-time cognitive load measurement (e.g., NASA-TLX with task decomposition) could provide sharper discrimination than retrospective coding allows.
